# The Characteristics and Clinical Evolution of Adolescents With Severe Chronic Pain in an Inpatient Multimodal Pain Programme

**DOI:** 10.1155/prm/6041949

**Published:** 2026-11-02

**Authors:** Guillaume Groffe, Aminata Ali, France Hirot, Adrien Naddaf, Anne Tonelli, Didier Bouhassira, Nathalie Godart, Chantal Stheneur

**Affiliations:** ^1^ Fondation Santé des Etudiants de France University Center for Adolescent and Young Adult Health Paris 75016 France; ^2^ UFR Simone Veil-Santé Université Versailles Saint-Quentin-en-Yvelines 78180 Montigny-le-Bretonneux France; ^3^ Fondation Santé des Etudiants de France University Center for Adolescent and Young Adult Health Paris 75014 France; ^4^ Equipe DevPsy Centre de Recherche en Épidémiologie et Santé des Populations (CESP) Hôpital Paul Brousse Université Paris-Saclay 94807 Villejuif France universite-paris-saclay.fr; ^5^ Groupe de Recherche en Médecine et Santé de l’Adolescent (GRMSA) Paris France; ^6^ Inserm U987 UVSQ Ambroise-Paré Hospital Paris-Saclay University Boulogne-Billancourt France universite-paris-saclay.fr; ^7^ Inserm U1178 UVSQ André Mignot Hospital Paris-Saclay University Versailles France universite-paris-saclay.fr

**Keywords:** adolescent, chronic pain, interdisciplinary rehabilitation pain programme, residential care

## Abstract

**Objectives:**

Around 5.1% of the French pediatric general population experiences severe chronic pain; it leads to school absenteeism, social isolation, depressed mood and anxiety. Only a few studies have been conducted on inpatient treatment for severe chronic pain. The aim of this research was to describe the characteristics of adolescents enrolled in an inpatient pain management programme within a residential therapeutic care center and their outcomes at discharge.

**Methods:**

We conducted a retrospective chart review of patients aged 11–19 who were hospitalized between 2010 and 2020.

**Results:**

We identified 196 hospitalizations for 195 adolescents. Their mean age was 15.4 ± 2.1 years, and 67.7% were female. Before admission, 92.9% experienced school absenteeism. At discharge, the mean number of weak‐ and strong‐opioid painkillers and/or chronic pain drugs prescribed was significantly reduced (mean difference = −0.70 ± 0.90, *p* < 0.001) and pain intensity was significantly lower than at admission on a 10‐point numerical scale (mean = 4.6 ± 2.5 at admission vs. 2.8 ± 3.3 at discharge (*p* = 0.01)). A school reintegration programme was documented for 167/196 (85.2%) hospitalizations.

**Discussion and Conclusion:**

Adolescents admitted to this inpatient programme presented with severe chronic pain, substantial school absenteeism, and a long history of healthcare utilization. At discharge, lower pain intensity, reduced painkiller prescriptions, reported improvements in social participation, and planned school reintegration were documented. These findings suggest that intensive multidisciplinary inpatient programmes may represent a valuable rehabilitation option for adolescents with complex chronic pain who remain significantly disabled despite specialized outpatient management and warrant further prospective evaluation.

## 1. Introduction

Chronic pain is defined in the ICD‐11 as pain that persists or recurs for more than three months and is influenced by biological, psychological and social factors [1]. It constitutes a major public health concern among children and adolescents. In a 2024 systematic review and meta‐analysis of 446 population‐based studies comprising 1,043,878 participants (52.0% female; mean age, 13.4 years [SD 2.4]), Chambers et al. estimated the global prevalence of chronic pain at 20.8% (95% CI, 19.2–22.4) with headache and musculoskeletal pain as the most prevalent conditions[2].

Chronic pain classified as severe is less common, with an estimated prevalence of approximately 5.1% in the pediatric general population [3].

The level of severity of pain is associated with significant functional and psychosocial consequences, including school absenteeism, social isolation, depression, anxiety [4] and increased healthcare costs [5].

In cases of severe impairment, when intensive outpatient interdisciplinary care is inefficacious, children with chronic pain need intensive care. In such cases, some countries (e.g., the United States, the United Kingdom, and Canada) provide access to day hospitals, while others offer residential care (e.g., Germany [6]).

Studies evaluating intensive interdisciplinary inpatient pain treatment programmes have consistently reported improvements in disability and other functional outcomes among adolescents with chronic pain [7, 8]. Benefits have also been observed in psychological functioning, quality of life, social functioning and school attendance [9, 10]. We found no published French study evaluating a residential programme combining intensive pain treatment and integrated schooling.

In France, the treatment trajectory for pediatric chronic pain is structured as a national, tiered network of services. In Paris and the surrounding areas, each center is certified by the Regional Health Agency [11].

For adolescents experiencing severe chronic pain and unresponsive to outpatient management, admission to a residential facility specialized in adolescent chronic pain treatment is offered [12]. Our team has developed an inpatient residential treatment programme of this type, coordinated by one of the authors (AT). This inpatient multimodal pain programme is combined with schooling delivered by the national education system (French Ministry of Education) and is located in the 16th district of Paris, at the Fondation Santé des Etudiants de France—a French Student Health Foundation (FSEF).

The aim of the present study was to describe adolescents hospitalized for severe chronic pain in a residential therapeutic care center and to report clinical, functional and educational outcomes documented between admission and discharge.

## 2. Materials and Methods

### 2.1. Procedures and Participants

We conducted a retrospective chart review including all patients consecutively admitted to the inpatient multimodal pain programme for chronic pain and pain‐related disability between 1 January 2010, and 31 December 2020. Due to COVID‐19–related restrictions, no new admissions were made between March and June 2020. Although balneotherapy activities were temporarily suspended and distance schooling was implemented between March and June 2020, the core components of the programme were maintained. Only two participants were affected by the pandemic, both being discharged slightly earlier than initially planned after having completed most of the programme. Given the very limited number of affected participants, the impact of the COVID‐19 pandemic on patient management and study findings was considered negligible.

Data were collected from the detailed, semistructured discharge medical report that is mandatory in France.

This report includes sociodemographic data, personal and familial medical history, chronic pain trajectory, the evolution of clinical symptoms during hospitalization (including pain at admission and discharge and past and current pain treatments) and finally treatment at discharge, care plan and schooling (see data collection below).

The study inclusion criteria were as follows: having chronic pain as the primary disabling condition, defined as pain that persists or recurs for more than 3 months, including both primary and secondary chronic pain; being hospitalized in the programme for the first time in the past 12 months of the period considered. The study exclusion criteria were incomplete or unusable chart reports, chronic pain was no longer the primary concern and refusing to participate (see Figure 1 flowchart).

**FIGURE 1:**
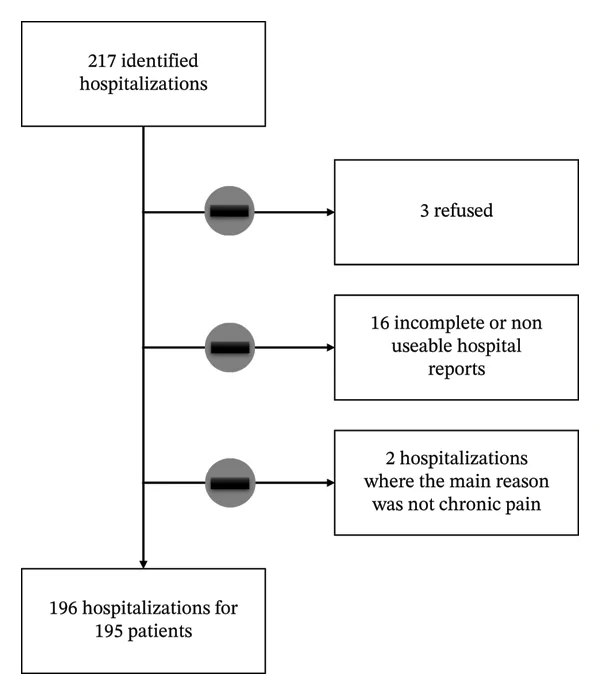
Flow chart of the study.

### 2.2. Therapeutic Setting

The inpatient multimodal pain programme was delivered in an inpatient unit comprising 30 beds with around 20 reserved for chronic pain patients. It was designed by a team led by Dr. Anne Tonelli in a FSEF residential therapeutic and schooling care clinic located in Paris (75,016) [12, 13].

The inpatient admission criteria were as follows: being between 11 and 19 years old, failure of an outpatient step‐by‐step increasing intensity care trajectory for pain (as recommended by the French National Authority for Health [HAS] [14] and also by the WHO [15]), and adolescents’ and parents’ willingness to join the programme.

The inpatient nonadmission criteria were as follows: a disability that did not allow patients to live in a community, individuals requiring intensive psychiatric care as a first‐line treatment owing to significant psychiatric symptoms, refusal of care by adolescents and/or adults and not being conversant in French.

The aims of hospitalization were to reduce the psychosocial consequences of chronic pain in order to promote resocialization, reschooling and a reduction in pain drug use [12, 13].

The admission process began with a first consultation, during which the care programme was presented to the adolescents and their parents by a medical doctor specialized in pain treatment and a nurse or a psychologist. Psychiatric evaluation was conducted when clinically indicated during hospitalization.

The adolescents were then given a tour of the clinic guided by a peer who was already hospitalized. To be admitted, the adolescents, and their parents in the case of minors, were to notify their agreement at the end of the first consultation.

Following admission, the adolescents followed the inpatient multimodal pain programme, which included weekly medical consultations with a practitioner specialized in pain treatment, every 2‐week individual psychotherapy focused on self‐esteem, the consequences of pain and secondary gains with a psychologist. Focusing on the psychological and emotional dimensions of chronic pain, group therapeutic mediation sessions were delivered several times a week as part of the multidisciplinary treatment programme. These sessions were structured group interventions led by healthcare professionals, using a specific activity or medium to facilitate discussion, reflection and interaction around health‐related experiences and concerns.

A consultation with a psychiatrist could be requested if necessary, and tailored care could be provided by nurses.

Drugs (painkillers and psychotropic drugs), as well as pain‐management aids or medical devices, were prescribed according to needs. A therapeutic education programme on the subject of chronic pain treatment and coping strategies was set up by the care team [12]. All the health professionals involved were trained on adolescent health and medicine. All of them shared the same goal: to help the patients become self‐sustained and in charge of their own illness.

In addition, adolescents participated in weekly rehabilitation sessions involving physiotherapy, occupational therapy, social work, psychomotor therapy (health professionals trained in psychomotor rehabilitation, prevention, health education and body‐oriented therapies) and adapted physical activity. Physiotherapy aimed to promote progressive re‐engagement in movement through physical reconditioning and muscular strengthening, while supporting the return to regular physical activity. Occupational therapy consisted of group‐based creative and practical projects designed to encourage social participation, peer interaction, autonomy and engagement in meaningful everyday activities. Adapted physical activity sessions were supervised by specially trained instructors and tailored to participants’ functional abilities and limitations.

In addition to the care provided by our therapists, we also offered a number of complementary activities such as Tai chi and yoga. Alongside this formal programme, there were many informal moments. Adolescents had a common room called the “Aquarium” at their disposal, where they could meet up and enjoy leisure and social activities. They were supervised by the educational team staff, who promoted socialization and fostered the creation of a peer group. An example of a typical weekly schedule provided to adolescents enrolled in the inpatient programme is presented in Table 1, illustrating the integration of therapeutic, rehabilitation and educational activities.

**TABLE 1:** Example of a typical weekly schedule within the inpatient multidisciplinary rehabilitation programme.

Time	Monday	Tuesday	Wednesday	Thursday	Friday
09:30–10:30	Schooling	Physiotherapy	Schooling	A.P.A.	Schooling
10:30–11:30	Pains specialist consultation	Schooling	Group therapeutic mediation	Schooling	Schooling
11:30–12:30	Schooling	Schooling	Schooling	Schooling	Group therapeutic mediation
**12:30–13:30**	**Lunch Break**	**Lunch Break**	**Lunch Break**	**Lunch Break**	**Lunch Break**
13:30–14:30	A.P.A.	Schooling	Schooling	Physiotherapy	Schooling
14:30–15:30	Schooling	Occupational therapy	Schooling	Schooling	Therapeutic education programme
15:30–16:30	Group therapeutic medication	Schooling	Physiotherapy	Schooling	Schooling
16:30–17:30	Nursing consultation	A.P.A.	Tai Chi	Yoga	Supervised social activities

*Note:* Lunch break is highlighted in bold to show how days are cut with this break.

The parents were actively involved in the treatment process. They had weekly consultations, most often by telephone, with the medical doctor. This not only gave them news about their teenager but it also helped them to understand the challenges of chronic pain. In addition to family interviews, the parents received therapeutic education about the biopsychosocial model of chronic pain.

The adolescents could leave the clinic for an hour every late afternoon, alone or with friends. They spent 2 days at home every other weekend. These free periods were provided to preserve familial and social inclusion.

A school was located in the hospital. The teachers came from the national education system (French Ministry of Education) and worked in close collaboration with the healthcare team. They provided adapted schooling on the premises in classrooms and not in the patients’ bedrooms [13]. The levels of school attendance were suited to the young people’s abilities. They could also sit compulsory school exams at the hospital. They followed a progressive timetable, which meant that they could return to their usual school at the end of their inpatient stay. Schooling thus became an extension of the treatment environment, as treatment and educational activities were highly integrated.

### 2.3. Data Collection

Based on the structure of the standardized discharge reports, the investigators (AA, AN, CS, and NG) developed a data extraction form to systematically collect the variables available in the medical records. The form was piloted on 10 reports and refined accordingly. Data extraction was then performed independently by two medical students. Discrepancies between the two datasets were identified by a biostatistician and reviewed against the original medical records by the first author. Any discrepancies were resolved by correcting the entry according to the source document.

Variables collected at admission and discharge are detailed in Tables 2 and 3. Admission data comprised demographic, family, medical, psychiatric, educational and pain‐related characteristics, as well as previous healthcare utilization and treatment history. Family‐level data were limited to information routinely documented in the medical records, including parental presence, parental employment status, number of siblings and social support measures. No additional family information was consistently available across participants. School absenteeism was classified a priori into four mutually exclusive categories: none, less than 50%, 50% or more, and complete absenteeism lasting at least three consecutive months. This study‐specific classification was developed to capture increasing levels of school absenteeism and to distinguish prolonged complete disengagement from school. The 3‐month threshold was informed by a previously published operational definition of temporary school dropout as absenteeism exceeding 3 months [16]. Socioeconomic status was assessed using the INSEE classification [17] based on parental occupation. Pain characteristics included pain duration, location, frequency, intensity and pain type. Pain type was classified into two categories: background pain, defined as the continuous chronic pain present between exacerbations, and episodic pain exacerbations, defined as temporary increases in pain intensity above the baseline chronic pain level. Because these episodes can have a substantial impact on patients’ daily functioning and quality of life, we considered it important to explore them separately. Pain intensity corresponded to the NRS score documented in the medical record. Pain intensity was assessed during the clinical evaluation at admission and discharge. Patients reported the mean intensity of both their background pain and pain exacerbations before admission and at discharge. As this was a retrospective chart review, no further standardized information regarding the assessment conditions was consistently available. At discharge, pain outcomes, treatment recommendations, functional outcomes and follow‐up arrangements were collected. Detailed definitions, sources, reporters and formats of all variables are provided in Tables 2 and 3.

**TABLE 2:** Variables collected at admission.

Domain	Variables	Source/reporter	Format
Demographic characteristics	Age, sex at birth	Medical record	Continuous, categorical
Family characteristics	Parental presence/alive status, parental employment status, number of siblings, social support measures, socioeconomic status (INSEE)	Medical record and family report documented in chart	Binary, count, categorical
Admission indications	Ambulatory care failure, school‐related difficulties, social impact, psychological impact, need for removal from home, postoperative care, other reasons	Medical record	Multiple binary variables
Medical history	Medical, surgical, gynecological, oncological, psychiatric history and treatments	Medical record	Binary/categorical
Psychiatric history	Anxiety, depressive symptoms, sleep disorders, suicide attempts, exposure to verbal, physical or sexual violence	Self‐report and/or documented diagnosis	Binary
Pain history	Age at pain onset, pain duration	Patient report documented in chart	Continuous
Healthcare utilization	Age at first pain specialist consultation, number of practitioners consulted, previous hospitalizations, referral source	Medical record	Continuous, count, categorical
Pain characteristics	Pain type (persistent pain and/or episodic pain exacerbations), pain location, pain frequency, pain intensity (NRS)	Patient‐reported average pain intensity during the admission/discharge clinical evaluation, documented in the medical record.	Categorical, continuous
Functional and educational impact	School absenteeism, individual care plan (PAI), mobility aids (splints, crutches, wheelchair)	Medical record	Ordinal, binary
Previous treatments	Pharmacological and nonpharmacological treatments	Medical record	Binary by category

**TABLE 3:** Variables collected at discharge.

Domain	Variables	Source/reporter	Format
Pain outcomes	Pain location, persistent pain intensity (NRS), episodic pain exacerbation intensity (NRS)	Patient‐reported average pain intensity during the discharge clinical evaluation, documented in the medical record.	Categorical, continuous
Clinical outcomes	Improvement in pain management, sleep and socialization	Clinician assessment documented in chart	Binary
Treatment and follow‐up	Discharge medications, recommendation for psychological and/or psychiatric follow‐up, referral destination	Medical record	Binary, categorical
Functional outcomes	Planned return to school, mobility aids (splints, crutches, wheelchair)	Medical record	Binary
Discharge characteristics	Discharge mode (planned discharge, family‐requested discharge, discharge against medical advice)	Medical record	Categorical

### 2.4. Statistical Analysis

Statistical analyses were performed using RStudio software (Version 1.2.5033). Analyses were primarily descriptive. Quantitative variables were summarized using means and standard deviations (SD) or medians and interquartile ranges (Q1; Q3), as appropriate, whereas categorical variables were described using frequencies and percentages. Exploratory bivariate analyses were performed to examine associations between patient characteristics and outcomes. Comparisons between categorical variables were conducted using Pearson’s Chi‐square test or Fisher’s exact test when expected cell counts were insufficient. Comparisons of continuous variables between two groups were performed using Student′s *t*‐test. Statistical significance was defined as a two‐sided *p* value < 0.05.

Several discharge outcomes were based on qualitative information documented in the medical records. Improvements in pain management, sleep, and socialization were recorded when explicitly reported by the patient and documented by the clinician in the discharge report. These variables were extracted as binary outcomes (improvement reported: yes/no) and are, therefore, presented descriptively.

### 2.5. Ethics

Approval for this study was obtained from the Paris‐Saclay Ethics Research Committee (number CER‐Paris‐Saclay‐2021‐001). As defined by French law, an information letter about the study was sent to all people hospitalized during the period considered, and they could refuse the use of their data. Information on these procedures is registered online on the public French directory Health Data Hub (N°F20210216170954).

## 3. Results

Of the 217 hospitalizations identified, 21 were excluded. The study sample consisted of 196 hospitalizations concerning 195 patients (Figure 1).

### 3.1. Description

A description of the sample in terms of family environment, economic situation and schooling is provided in Table 4. The mean age was 15.4 ± 2.1 years, and the sex ratio was 0.48 (M/F). Among the parents, five fathers and one mother were deceased. The report indicated that four of the parents had not been living with the adolescent or that they had not been in contact for several years. One patient had been adopted.

**TABLE 4:** Sociodemographic characteristics and family environment of the adolescents hospitalized for chronic pain.

For a total 196 hospitalizations	*N*	%
Patient’s gender		
Female	132	67.3
Male	64	32.7
Family environment		
Separated parents	84	40.7
Presence of siblings	171	87.2
Socioeconomic environment		
Unemployed father	23	11.7
Working father	173	88.3
Self‐employed skilled workers, shopkeepers and small business owners	20	11.6
Executives and higher intellectual professions	31	15.8
Intermediate professions	38	19.4
Clerical and service workers	21	10.7
Manual workers	29	14.8
Unspecified	34	17.3
Unemployed mother	40	20.4
Working mother	156	79.6
Self‐employed skilled workers, shopkeepers and small business owners	7	3.6
Executives and higher intellectual professions	23	11.7
Intermediate professions	61	31.1
Clerical and service workers	44	22.4
Manual workers	5	2.6
Unspecified	16	8.2

### 3.2. Pain Management Trajectory

The mean age at pain onset was 11.1 ± 3.8 years, with a first specialized consultation in a pain management center at a mean age of 13.8 ± 2.4 years. Half of the adolescents had consulted at least five different medical practitioners [Q1 = 4; Q3 = 13] and had one hospitalization for pain [Q1 = 0; Q3 = 2].

Before admission, 186/196 (95%) of the hospitalization reports mentioned that the adolescents were taking pain drugs. The mean number of prescriptions of weak‐ and/or strong‐opioid painkillers and/or chronic pain drugs was 1.2 ± 0.7. Weak opioids were prescribed in 50% of cases, strong opioids in 9.6% and chronic pain drugs (amitriptyline, pregabalin and duloxetine) in 64%.

Regarding the nondrug management of the patients’ chronic pain, 57/196 (29.1%) reports stated that the adolescents had received physiotherapy, occupational therapy, balneotherapy, TENS or osteopathy. Alternative medicines were mentioned in 29/196 cases: 1 homeopathy, 7 acupuncture and 21 others, including magnetism, auriculotherapy kinesiology, Reiki and oxygen therapy. Equine‐assisted therapy had been prescribed for one patient.

During clinical examination on admission, the mean pain intensity according to a 10‐point numerical scale was 4.5 ± 2.5. At admission, 187/196 (95.4%) reports mentioned episodic pain exacerbations associated, in most cases, with background chronic pain, 175/196 (89.3%). The mean persistent pain intensity measured on a 10‐point scale was 5.13 ± 2.16, and the mean paroxysmal pain intensity measured on a 10‐point scale was 8.60 ± 1.53. Musculoskeletal pain was the most common pain type reported (102/196, 52.0%), followed by headaches (including migraine and tension‐type headache) (98/196, 50.0%) and abdominal pain (66/196, 33.7%).

### 3.3. Medical History

In the discharge medical report, personal medical history included a chronic disease for 111/196 (68.9%) of the hospitalizations, surgical history related to pain for 50/196 (25.5%), surgery not related to pain for 29/196 (14.8%) and cancer in remission for 11/196 (7.0%).

The most frequent chronic diseases were asthma 22/111 (19.8%), Ehlers–Danlos syndrome 8/111 (7.2%) and juvenile idiopathic arthritis 6/111 (5.4%). Allergy was reported in 10/111 (5.1%).

More than one‐quarter of the reports (53/196, 27.0%) mentioned a history of self‐reported psychological symptoms or symptoms diagnosed by a physician, including 38/196 (19.4%) with depressive symptoms and/or anxiety for 23/196 (11.7%) reports. Four patients had already attempted suicide (2.0%).

Previous psychological treatment was found for 98/196 (67.6%) hospitalizations and psychiatric treatment for 41/196 (28.5%).

A history of chronic pain was present among first‐degree family members for 84/196 (42.9%), a chronic disease for 89/196 (45.4%) and a past psychiatric history for 38/196 (19.4%).

### 3.4. Disabilities

Almost half of the sample was in general lower secondary school, 93/196 (47.4%), and the other half in upper secondary school, 92/196 (46.9%). Five (2.5%) patients were enrolled in vocational courses. School absenteeism was reported in 182/196 (92.9%) reports.

Sleep disorders were present in 108/196 (55.1%) reports, and social isolation was mentioned in 52/196 (26.5%) reports.

Because of their pain, 35/196 (17.9%) patients were provided with pain‐management aids at the time of admission.

#### 3.4.1. Pain at Discharge

The mean length of stay was 6.4 ± 4.9 months.

Only 189/196 (96.4) reports mentioned a pain assessment at the end of hospitalization. At the time of discharge, pain was significantly less intense than at admission (mean = 4.6 ± 2.5 [admission] vs. 2.8 ± 3.3 [discharge] on a 10‐point scale [Student′s *t*‐test, *p* = 0.01]). Furthermore, we observed a significant decrease in reported background pain: 134/196 at discharge versus 170/196 at admission, amounting to a decrease of 21.2% (Pearson’s Chi‐square test, *p* < 0.001). Reports indicated a significant decrease in self‐reported episodic pain exacerbations: 183/196 (93.4%) at admission versus 153/196 (78.1%) at discharge (Pearson’s Chi‐square test, *p* = 0.002). We also observed a significant decrease in the intensity of the background pain with a mean difference of −0.8 ± 1.8 (Student′s *t*‐test, *p* < 0.001) on the 10‐point scale (Figure 2) and in the intensity of episodic pain exacerbations with a mean difference of −0.9 ± 1.7 (Student′s *t*‐test, *p* < 0.001) on the 0‐ to 10‐point scale (Figure 2).

**FIGURE 2:**
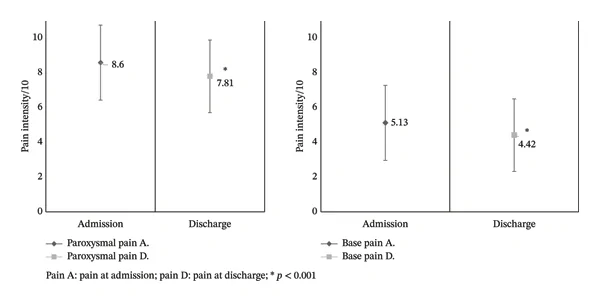
Evolution of persistent pain intensity and paroxysmal pain intensity between admission and discharge.

In 164/196 reports (83.7%), an improvement in the patients’ pain management was reported by the patient to their physician.

#### 3.4.2. Evolution of Treatment Following the Programme

At discharge, compared to admission, we observed a decrease in the number of patients taking pain drugs 34/196 (0.17%—admission) versus 5/196 (0.03%—discharge), Pearson’s Chi‐square test (*p* = 0.016) and a significant decrease (mean difference = −0.70 ± 0.90 [Student′s *t*‐test, *p* < 0.001]) in weak‐ and/or strong‐opioid painkillers and/or prescribed drugs for chronic pain.

#### 3.4.3. Evolution of Disabilities Associated With Chronic Pain at Discharge

The following outcomes reflect patient‐reported improvements documented in discharge reports and are presented descriptively.

Full‐day school reintegration was reported in 167/196 (85.2%) reports, including 75 (38.2%) with adapted schooling (9 in a psychiatric FSEF residential care center with schooling). Only 2/196 adolescents (1.0%) did not return to school. For 26/196 reports (13.3%), hospitalization in an acute care ward for a medical or psychiatric condition before completion of the programme prevented the collection of complete outcome data. Sleep improved for 63/82 (76.8%) and social isolation decreased for 66/81 (81.5%). The use of pain‐management aids was descriptively lower at discharge, but the limited number of observations prevented statistical testing. At discharge, two adolescents (1.0%) needed a wheelchair, 2/196 (1.0%) used two crutches, 2/196 used a single crutch (1.0%) and 2/196 (1.0%) required a brace.

During hospitalization, a psychiatric evaluation was requested for 96/196 patients (49.0%), either for patients with a previously established psychiatric diagnosis and ongoing treatment or for patients identified by the multidisciplinary care team as potentially presenting with a psychiatric condition requiring specialist assessment. At discharge, ambulatory psychiatric care was recommended for 74/196 (37.8%) and ambulatory psychological care for 103/196 (52.6%). An antidepressant in the discharge prescription was found in 38/196 (19.4%) reports and anxiety and/or sleep drugs in 42/196 (21.4%).

The discharge mode was mostly in agreement with the medical team for 163/196 (83.2%) hospitalization reports; for 28 (14.3%), discharge was early at the family’s request, and for 5/196 (2.55%), discharge was against medical advice.

Of the 196 discharge reports reviewed, 161 (82.1%) mentioned discharge home, whereas 35 (17.9%) documented referral to another hospital unit. Among these referrals, 24 (68.6%) concerned psychiatric care, including 15 (42.9%) to an acute psychiatric ward and 9 (25.7%) to an FSEF residential care center with integrated schooling [18]. Follow‐up in a pain management center was planned in 176 reports (89.8%), while five (2.6%) indicated follow‐up by a general practitioner; follow‐up was not specified in 15 reports (7.7%).

## 4. Discussion

This study reports for the first time the characteristics and clinical evolution of a large sample of French adolescents with severe chronic pain who required hospitalization in an inpatient multimodal pain programme combining therapy and schooling in a residential care center. Before admission, 192 patients (98.0%) had received ambulatory care from specialized teams and 4 (2.0%) from specialized physicians. These interventions had proved insufficient, justifying the need for intensified care through inpatient treatment.

Within a few weeks of inpatient care, patients showed significant reductions in pain intensity and painkiller use. In parallel, medical records documented improvements in social participation and school engagement, as well as the initiation of psychological follow‐up for some adolescents. These findings suggest that inpatient care may support the rehabilitation process in adolescents with chronic pain.

The characteristics of pain at admission are in line with those reported in other pediatric inpatient and outpatient samples [19–26]. There is not much difference in pain intensity between inpatient and outpatient samples [27–29], but what is characteristic of inpatient populations is the intensity of disability at admission. This suggests that healthcare professionals need to work not only on pain reduction, but also on the adolescents’ perception of themselves as disabled. For this purpose, programmes such as ours worldwide have been designed around multidisciplinary care and work with the family [6, 19, 24, 29–31]. These programmes, ours included, are based on somatic care, physiotherapy, school participation, psychological care and talking groups, accompanied by occupational therapy. Emphasis has been on psychocorporeal methods and rehabilitation, as recommended by the WHO [15].

More importantly, all authors have reported working on reducing painkiller treatments, which can be inappropriate or unnecessary, and promoting nonpharmacological techniques, such as relaxation and occupational therapy.

Our study specifically highlights patterns of pharmacological treatment before hospitalization and at discharge, with particular attention to weak and strong opioid painkillers and chronic pain drugs. This is important, as chronic pain during adolescence has been shown to lead to opioid misuse in adulthood [20]. Educating young people about painkiller use during adolescence could help limit opioid misuse in adulthood. Our results show a large number of opioid prescriptions (64%) for these adolescents with chronic pain.

This percentage is very high, probably because our population consisted of patients who had failed to respond to previous therapies, prompting physicians to explore additional painkiller options. However, opioids are not recommended by the WHO [15]. Opioid prescribing practices should be interpreted cautiously, as in France the use of opioids for adolescents with chronic pain remains relatively uncommon, including in specialized pain clinics [32].

Few data are available regarding psychiatric assessments and psychotropic prescribing practices in adolescents hospitalized for chronic pain. Our study contributes additional information on these aspects of care. Although 41 patients were already under psychiatric care prior to hospitalization, the large number of psychiatric consultations and psychiatric drug treatments prescribed during hospitalization suggested an underdiagnosis and undertreatment of psychological comorbidities associated with chronic pain among these adolescents, despite the fact that pain‐related cognitions or emotions have shown higher correlations with disability than pain intensity [33, 34]. The high prevalence of psychiatric comorbidities observed in our cohort is consistent with the findings of the systematic review and meta‐analysis by Dudeney et al. [35], which highlight the frequent coexistence of anxiety and depressive disorders in adolescents with chronic pain. The slightly higher prevalence of psychiatric comorbidities observed in our cohort may be explained by a recruitment bias, as our multidisciplinary programme preferentially receives adolescents with complex clinical presentations and significant school disengagement. Accordingly, the substantial number of referrals to specialized psychiatric services at discharge likely reflects both this recruitment pattern and the identification of psychiatric conditions requiring dedicated mental health care.

The main challenge of the programme was to enable adolescents to resume an ordinary life, including school attendance, social activities, and everyday functioning, as quickly as possible.

School absenteeism is one of the most tangible manifestations of functional disability in pediatric chronic pain and is increasingly recognized as a key indicator of treatment outcome. School attendance is essential not only for educational attainment but also for psychosocial development, socialization and identity construction during adolescence[36, 37]. Recent studies have shown that adolescents with chronic pain frequently experience substantial school absenteeism, and that persistent absenteeism is associated with poorer long‐term outcomes and greater functional disability[38, 39]. This disability was considerable in the present cohort compared to the mean rate of absenteeism in the French general population [40], among French ambulatory pediatric patients with chronic pain [32], or among adolescents in a similar German center [31]. The strength of our programme in the FSEF hospital is that it includes schooling with the involvement of the French Ministry of National Education [12], which enables schooling to be gradually resumed in cases where absenteeism was high before hospitalization. If required, readaptation to school can be facilitated through discussions between the care team and the teaching staff, with the option of sitting school examinations. This was all the more challenging as the level of school absenteeism was found to be very high. Work on academic support is often a major task, particularly because young people’s feelings about their own incompetence can increase absenteeism. The integration of educational support as a core therapeutic component of our multidisciplinary programme may partly explain the longer mean hospitalization duration compared with other studies, as it not only prevents academic delay but also supports the gradual restoration of self‐efficacy and functional reintegration into school and social life. Finally, a school reintegration plan was documented at discharge for 85.3% of adolescents. School attendance is widely recognized as a major functional outcome in pediatric chronic pain. The high proportion of adolescents leaving the programme with a documented school reintegration plan highlights the central place of school participation within chronic pain management. This emphasis on school reintegration is consistent with previous findings from a French ambulatory cohort, which identified school absenteeism as an important marker of disease burden in adolescents with chronic pain[32].

Inpatient multidisciplinary programmes are intended for a small subgroup of adolescents with chronic pain who experience severe functional impairment despite specialized outpatient management. Beyond pain reduction, their objective is to promote rehabilitation through intensive and coordinated interventions targeting physical activity, psychological functioning, social participation, and school attendance. Most adolescents included in our programme were referred by specialized pediatric pain services after insufficient improvement with outpatient management, including multidisciplinary day‐hospital programmes when available. The high rates of previous treatment failures, healthcare utilization, and school absenteeism observed in our cohort support the interpretation that these adolescents represented a particularly vulnerable and treatment‐resistant population.

## 5. Limitations and Strengths

This study has several limitations. First, it was a retrospective single‐center study based on medical records from a single clinical team and did not include a comparison group. Consequently, the observed changes cannot be attributed solely to the treatment programme. In addition, randomized controlled trials involving adolescents with disabling chronic pain raise important ethical and practical challenges, often requiring multicentre collaboration [41]. The retrospective study design limited analyses to routinely documented clinical variables. Validated quality‐of‐life measures were not routinely administered within the programme and were, therefore, unavailable for analysis. Our cohort represents one of the largest published samples of adolescents hospitalized for treatment‐resistant chronic pain. All discharge reports followed a standardized semistructured format consistent with the recommendations of the French National Authority for Health (HAS) ([42], s. d.; [43]) and were completed by clinicians specialized in adolescent chronic pain. This likely contributed to the consistency of data collection across patients. Future studies would benefit from a prospective design, the inclusion of a comparison group, and the use of validated outcome measures incorporating both patient‐reported and objective functional assessments.

Although pain intensity alone does not fully capture rehabilitation outcomes in adolescents with chronic pain, it remains one of the most frequently reported outcome measures and was assessed at admission and discharge in previous inpatient studies. Future research should complement pain intensity measures with broader assessments of functioning, participation, and quality of life in order to provide a more comprehensive evaluation of treatment outcomes.

## 6. Conclusion

Our study provides a detailed description of adolescents with treatment‐resistant chronic pain who were managed in an inpatient multimodal programme and of their outcomes at discharge. Beyond pain intensity, the findings emphasize the importance of assessing functional and psychosocial domains, particularly school participation. Although the retrospective design precludes any conclusions regarding treatment effectiveness, the observed outcomes support further investigation of intensive multidisciplinary programmes for adolescents with complex chronic pain. Prospective studies with longer follow‐up and standardized outcome measures are warranted.

**Nomenclature**
FSEF: Fondation Santé des Etudiants de France (French Student Health Foundation)
HAS: Haute Autorité de Santé (French National Institute for Health)
IASP: International Association for the Study of Pain
INSEE: Institut National des statistiques et des Etudes Economiques (National Institute for Statistics and Economic Studies)
NRS: Numerical Rating Scale
PAI: Projet d’Accueil Individualisé (Individualized Care Planning at School)
SD: standard deviations
TENS: Transcutaneous Electrical Nerve Stimulation
WHO: World Health Organization

## Author Contributions

This study was designed by Aminata Ali, France Hirot, AD, Anne Tonelli, Nathalie Godart and Chantal Stheneur. The data collection was performed by Guillaume Groffe. The data were analyzed by Guillaume Groffe and Aminata Ali, and the results were critically examined by all authors. Guillaume Groffe had a primary role in preparing the manuscript, which was edited by Didier Bouhassira, Nathalie Godart and Chantal Stheneur.

## Funding

This work was supported by Apicil Fondation, 1493.20.

## Disclosure

All authors have approved the final version of the manuscript and agreed to be accountable for all aspects of the work. Apicil Fondation does not have any impact or influence over the design or outcome of the study.

## Conflicts of Interest

The authors declare no conflicts of interest.

## Data Availability

The data that support the findings of this study are available on request from the corresponding author. The data are not publicly available due to privacy or ethical restrictions.
